# The use of imepitoin (Pexion™) on fear and anxiety related problems in dogs – a case series

**DOI:** 10.1186/s12917-017-1098-0

**Published:** 2017-06-13

**Authors:** Kevin J. McPeake, Daniel S. Mills

**Affiliations:** Animal Behaviour, Cognition and Welfare Group, School of Life Sciences, University of Lincoln, Lincoln, Lincolnshire UK

**Keywords:** Anxiety, Dog, Drug, Fear, GABA, Imepitoin

## Abstract

**Background:**

Fear and anxiety based problems are common in dogs. Alongside behaviour modification programmes, a range of psychopharmacological agents may be recommended to treat such problems, but few are licensed for use in dogs and the onset of action of some can be delayed. The low affinity partial benzodiazepine receptor agonist imepitoin (Pexion™, Boehringer Ingelheim) is licensed for treating canine epilepsy, has a fast onset of action in dogs and has demonstrated anxiolytic properties in rodent models. This case series reports on the use of imepitoin in a group of dogs identified as having fear/anxiety based problems. Twenty dogs were enrolled into the study, attended a behaviour consultation and underwent routine laboratory evaluation. Nineteen dogs proceeded to be treated with imepitoin orally twice daily (starting dose approximately 10 mg/kg, with alterations as required to a maximum 30 mg/kg) alongside a patient-specific behaviour modification plan for a period of 11–19 weeks. Progress was monitored via owner report through daily diary entries and telephone follow-up every two weeks. A Positive and Negative Activation Scale (PANAS) of temperament was also completed by owners during baseline and at the end of the study.

**Results:**

The primary outcome measure was average weekly global scores (AWG) from the owner diaries. Average weekly reaction scores (AWR) for each type of eliciting context was used as a secondary outcome. Seventeen dogs completed the trial. Treatment with imepitoin alongside a behaviour modification programme resulted in owner reported improvement with reduced AWG and reduced AWR for anxiety across a range of social and non-social eliciting contexts including noise sensitivities. Significant improvement was apparent within the first week of treatment, and further improvements seen at the 11 week review point. There was a significant reduction in negative activation (PANAS) with 76.5% of owners opting to continue imepitoin at their own expense after completion of the study.

**Conclusions:**

This study provides initial evidence indicating the potential value of imepitoin (Pexion™) alongside appropriate behaviour modification for the rapid alleviation of signs of fear and anxiety in dogs. Further research with a larger subject population and a placebo control would be useful to confirm the apparent efficacy reported here.

**Electronic supplementary material:**

The online version of this article (doi:10.1186/s12917-017-1098-0) contains supplementary material, which is available to authorized users.

## Background

Fear and anxiety-related problems in dogs are common. In the People’s Dispensary for Sick Animals Pet Animal Welfare report [1] (2011) 82% of owners reported their dog was afraid of ‘something’. Another study [2] found that 25% of owners considered their dogs to be fearful of noises, but 49% reported at least one behavioural sign suggestive of fear when exposed to loud noises. The Association of Pet Behaviour Counsellors in the UK [3] report that 8% of canine cases were referred for a specific fear or phobia, 6% for owner-absent problems (which includes “separation anxiety”) and 64% for aggressive behaviour towards people and dogs (the extent to which fear or anxiety played a role in these cases was not stated). In the US, a study [4] found 10.3% of cases had a specific fear, anxiety or phobia, 14% separation anxiety and 22% fear-related aggression towards people.

Both fear and anxiety can occur in the context of the perception of increased threat to the individual [5]. Whereas fear occurs in response to the presence of a specific aversive trigger, anxiety develops when an animal anticipates a negative outcome such as in a location where they previously encountered an aversive trigger [6, 7]. In a novel or unpredictable environment, or in a situation of ambiguous threat, a dog may experience anxiety and uncertainty due to a conflict of approach and avoidance tendencies [8]. Dogs may also experience anxiety associated with the departure of the owners. These may arise from fear and anxiety related associations (e.g. fear of isolation, fear of stimuli occurring in the context of being separated) as well as problems related to separation from an attachment figure (PANIC sensu Panksepp, [9]). Different neurochemical networks are thought to underpin these different forms of anxiety [10, 11]. A further distinction may also be made between social and non-social stimuli that trigger a response [12].

The affective state of fear and anxiety can operate at the level of: a specific emotional reaction (i.e. in response to a specific aversive trigger); a mood change (longer lasting emotional changes that bias behaviour and cognition occurring in response to a series of related aversive events); or as a feature of temperament (behavioural predispositions arising from the interaction of genetic and early experiential factors). One method developed for assessing aspects of temperament in dogs is the Positive and Negative Activation Scale (PANAS) [13], a reliable and valid measure of positive activation/affect (mediates behavioural approach and neophilia) and negative activation/affect (mediating behavioural inhibition, withdrawal and avoidance (fear-anxiety)). Anxious emotional reactions, moods and temperament can all be problematic for an owner and are a cause for welfare concern.

Fear and anxiety related problems in dogs are principally resolved using behaviour modification techniques such as desensitisation and counter-conditioning (operant and classical) [14]. Various psychopharmacological agents have been suggested as potentially useful adjuncts in such cases, [15] including: tri-cyclic antidepressants (TCAs) e.g. amitriptyline [16] and clomipramine [17, 18]; tetra-cyclic antidepressants e.g. mirtazapine [14]; selective serotonin reuptake inhibitors (SSRIs) e.g. fluoxetine [19, 20]; monoamine oxidase inhibitors (MAO-Is) e.g. selegiline [21]; progestins e.g. megestrol acetate [22]; anticonvulsants e.g. phenobarbital [23]; benzodiazepines e.g. diazepam [24] and alprazolam [25]; alpha-2 adrenergic receptor agonists clonidine [26] and dexmedetomidine [27].

However, there are few psychopharmacological agents licensed for use in dogs. In the European Union (EU), where a licensed drug is not available, veterinary surgeons should follow the prescription cascade as outlined in the European Union Veterinary Medicines Directive (2001/82) [28]. Although this allows the use of the benzodiazepines listed above because they have a human licence, the prescription cascade regulations indicate that for a different condition in the same species should be given preference. In the United States (US), where a veterinary formulation does not exist informed consent can be obtained from a client to use human formulations “extra-label”.

At the time of writing, clomipramine (Clomicalm, Novartis – EU and US), selegiline (Selgian, CEVA - EU; Anipryl, Zoetis, US), the progestogen megestrol acetate (Ovarid, Virbac – EU), fluoxetine (Reconcile – not currently available) and most recently dexmedetomidine (Sileo, Zoetis) all have veterinary licenses for dogs.

The effects of using medications such as SSRIs and TCAs can typically take around 3–5 weeks to become apparent [15]. Benzodiazepines have a faster onset of action [15, 29], but no benzodiazepines are licensed in any form for use in dogs in the EU nor available as veterinary formulations in the US.

Benzodiazepines exhibit their effects by binding to a specific benzodiazepine binding site on the inhibitory neurotransmitter GABA (ɣ-aminobutyric acid) receptor and they can exhibit anxiolytic properties [30]. One retrospective study in dogs explored the anxiolytic properties of diazepam as reported by owners, where the response was variable, where 53% of owners discontinued diazepam therapy due to lack of efficacy and 58% due to adverse effects [24]. In the same study in the group of dogs classed as having ‘thunderstorm phobia’ 100% of owners classed the treatment as effective. Dosages of diazepam varied widely in this study, which is important as the effects of benzodiazepines are dose dependent with moderate doses often needed for anxiolytic effects and higher doses more likely to cause adverse effects such as ataxia [15]. In addition, tolerance can develop with the use of benzodiazepines [15, 31]. However, as benzodiazepines can be highly effective there is value in further exploring the safety and reliability of using this class of drugs as anxiolytics in dogs.

Imepitoin (Pexion™, Boehringer Ingelheim) is a low affinity partial benzodiazepine receptor agonist [32, 33] licensed in the EU for the reduction of the frequency of generalised seizures due to idiopathic epilepsy in dogs [34]. When used in dogs, imepitoin appears to be well tolerated and safe [35, 36]. Pharmacokinetic studies in dogs show imepitoin has a fast onset of action of around 2–3 h after single oral dosing [37]. Fear and anxiety are common behavioural co-morbidities in canine epileptic patients [38]. During the development of imepitoin for the treatment of idiopathic epilepsy in dogs, it was reported anecdotally that some owners wanted to continue using imepitoin due to reported improvements in their dog’s behaviour even when seizure frequency was unaffected [39]. In a variety of experimental rodent models, imepitoin has demonstrated anxiolytic properties [39–41]. In addition, being a low affinity partial agonist, the likelihood of developing reduced efficacy related to tolerance and potential for abuse are lower which may offer advantages over full agonist benzodiazepines [39].

The aim of the current study was to undertake an initial investigation of the potential value of imepitoin alongside an individualised behaviour modification programme for the treatment of a range of anxiety and fear related behaviour problems in dogs through a carefully monitored case series.

## Methods

### Subjects

Cases were recruited following a local publicity campaign via referral from the owner’s regular veterinary surgeon. Clients were offered a free behaviour consultation, blood test, trial medication and follow up for a period of up to three months. Potential cases were screened using a series of inclusion and exclusion criteria (Table 1).

**Table 1:** Inclusion and exclusion criteria

Inclusion criteria	Exclusion criteria
At least 6 months of age Greater than 5 kg bodyweight Behavioural complaint related to fear-anxiety as diagnosed by a veterinary behaviourist Incidences of behavioural complaint apparent at least twice a week Willing to attend a behaviour consultation at the University of Lincoln Willing to commit to giving medication for up to a 3 month period if medication deemed to be of potential benefit to the case (owner has option of withdrawing from study at any time) Willing to have a blood sample taken from their dog for routine laboratory evaluation (at no expense to owner), prior to use of any medication Willing to complete all study paperwork including a daily diary referring to the animal’s behaviour problem and more global behaviour	Lactating or pregnant bitch Male breeding dog Dogs receiving phenobarbital treatment for epilepsy Any current uncontrolled medical problem Severely impaired hepatic function Severe renal disorders Severe cardiovascular disorders Significant family related risk factors affecting risk to individuals from the dog’s behaviour identified (e.g. toddlers in the home in the case of aggressive behaviour towards people; aggression between dogs within home)

To be considered for the study, 4 initial documents had to be completed: a veterinary referral form to be completed by their referring vet and returned with a full medical history; the University of Lincoln Animal Behaviour Clinic standard client questionnaire; a Positive and Negative Activation Scale (PANAS); an individualised diary (Additional file 1) to be completed to provide one week of baseline data for their dog’s reaction in eliciting context(s) established through a telephone discussion. The diary was adapted from The Lincoln Sound-Sensitivity Scale [10] to relate more broadly to recognisable signs of fear and anxiety in contexts other than noises. To do this an on-line forum of pet behaviour counsellors [42] was asked to provide signs they would attribute to fear and anxiety in dogs. As a result of this three signs were added to the diary – yawning; licking lips; moving away. This gave 20 specific signs with an additional ‘others’ category. The ‘frequency’ score was replaced by an ‘Event Occurrence’ box which could be marked ‘yes’ or ‘no’. The severity score range was changed from ‘0–5’ to ‘1–5’. A total score for the dog’s reaction to that eliciting context on that occasion, could then be calculated by totalling each severity score for each sign/category (maximum score = 105). Examples of eliciting contexts included: encounters with strangers; noises; dogs etc. The same diary format was used during baseline and the follow up period to monitor the dog’s reactions (‘Event occurrence’ and ‘Severity’) based on the owner’s observation and scoring. For the single dog with separation related problems (Case 17), video footage was taken weekly whilst the dog was home alone and reviewed by the owner in order to complete the diary scoring. Of the initial 28 owners sent this information, 7 failed to return the complete paperwork and one dog was euthanized before the consultation for unrelated reasons. This left 20 dogs with suspected fear and anxiety related problems.

The clinician in immediate charge of the case (KM) under the supervision of a European and RCVS recognised specialist in behavioural medicine (DM) assessed all the documents completed at the enrolment stage.

### Behaviour consultation

Each of the 20 dogs in the initial group was brought to the University of Lincoln Animal Behaviour Clinic for a behaviour consultation, typically lasting 2–3 h. The approach used to establish a diagnosis relating to fear-anxiety adopted the systematic and scientific evaluation of the four key components of emotion (as described by Mills et al.*,* [10]) relating to context, physiological arousal, behavioural tendencies and communicative elements.

### Clinical examination and blood sampling

During the consultation, a full clinical examination was performed on each dog and bodyweight was measured. Jugular venepuncture was performed to obtain a complete blood count, serum biochemistry, thyroid panel (fT4, TT4, cTSH) and basal cortisol values for each dog.

### Behaviour modification programmes

Given the wide range of presenting problems, it was not desirable to standardise the treatment protocols for each dog. Behaviour modifications were tailored to the individual, taking into account the dog, the presenting problems, the owner and other factors relating to the dog’s specific environment. Advice was given during the consultation on techniques, which were demonstrated where necessary, and owners were provided with an aide memoire of key points, followed up by a full written report within 7 days of the consultation.

### Use of imepitoin

As imepitoin is licensed for use in dogs at a dose range of 10-30 mg/kg twice daily, all cases were commenced on a dose around 10 mg/kg twice daily. During the study, and depending on the individual’s progress, dose alterations were made. Dose changes were made at intervals of no less than two week periods. The rationale for increasing the dose was twofold: 1) if there was no apparent improvement at the previous dose; 2) if improvement at the previous dose had plateaued and the behaviour problem was not resolved to the satisfaction of the owner and behaviour clinicians.

### Monitoring during follow-up period

During the follow up period, owners completed an eliciting context diary sheet every time their dog was exposed to an eliciting context identified in the baseline period, and these were submitted to the investigators every two weeks. These diaries were scored and a telephone call to the owner was arranged to review progress.

### End of study

All cases used in the final analysis were followed for at least 3 months including the one week of baseline monitoring before reaching the ‘decision point’ (this varied between 11 to 19 weeks depending on the availability of the owner to complete a telephone survey at the end of the study). At this time owners were asked to complete a second PANAS and questioned about various aspects of their experience of using imepitoin including: ease of administration; satisfaction with overall treatment success; whether they would use imepitoin at their own expense (and if so, tactically or continuously). They were also given the clinician’s opinion on using imepitoin for their dog in the future and were offered further advice on behaviour modification as necessary. In those owners continuing imepitoin responsibility for prescribing imepitoin was passed to the referring veterinary surgeon. Imepitoin can be stopped abruptly, however for those owners stopping imepitoin, guidelines were given for weaning which was typically reducing each dose by 50% for 2 weeks before stopping completely

### Statistical analysis

The data were analysed informally for evidence of a relationship between imepitoin dosing and owner report of changes in behaviour with statistical analysis undertaken using Minitab 17. Normality of distribution of data were assessed using Anderson-Darling normality tests. Effect sizes were calculated for all assessments of difference using Cohen’s d [43].

#### Effects on reactions

Two main metrics were used to monitor efficacy on fear-anxiety reaction scores: average weekly fear-anxiety reaction scores (AWR) and average weekly global fear-anxiety scores (AWG). The equations were developed specifically for this study to use the owner’s diary entry scores to assess response and control for the number of eliciting contexts.

In addition, given that the eliciting contexts did not occur every week, we adopted a method of conservative data imputation in order to avoid having missing scores. Imputations for responses in weeks when the eliciting context did not occur in a given week were made using the following rules: if a week of data was missing during the follow up, the average of the weeks on either side was taken and imputed (i.e. the value used for that week was estimated from the average of the week before and afterwards); if in the final week of the follow up period, there was no eliciting context, the last value obtained before this was imputed. This method probably provided a conservative estimate of the effect, potentially underestimating any effect, since it referred back to an earlier stage of treatment. At all the time points analysed (baseline, week 1, week 11 and decision point): for the primary measure of interest, average global weekly scores, 3 imputations were made out of 68 entries (3/68 = 4.4%); for the measure of secondary interest, average weekly reactions, 28 imputations were made out of 140 entries (28/140 = 20%). All these data related to an absence of the eliciting context in a given week (e.g. a week where there was no exposure to an eliciting context recorded by the owner) and not lost data from recorded responses.

Statistical analysis was stratified with AWR and AWG as the primary outcome measures and subsequent analyses post hoc based on the significance of the primary measures. As there were 2 measures a Bonferroni correction was applied to AWR and AWG only as to apply it to all post hoc calculations would risk a type II error.

##### Average weekly reactions scores

The average weekly reaction score (AWR) (maximum score 105) was calculated for each dog for each eliciting context as shown in the example below for a dog with noise sensitivities:

$$ AWR\ \left( individual\  noise\right)=\frac{Sum\  of\  diary\  scores\  for\  that\  individual\  noise\  that\  week}{Number\  of\  exposures\  to\  that\  individual\  noise s\  that\  week} $$

$$ AWR\ (noises)=\frac{Sum\  of\  all\  AWR\ \left( individual\  noises\right)\  that\  week}{Number\  of\  different\  individual\  noises\  exposed\  to\  that\  week} $$

Similar equations were used to calculate AWR scores for dogs with social fear-anxiety and also non-social fear-anxiety. To compare differences in the AWR in dogs between eliciting contexts grouped into noise sensitivities, social fear-anxiety and non-social fear-anxiety (excluding noise sensitivities at specific time points (week 1, week 11, decision point) whilst on treatment through the study, paired sample t-tests were used. The accepted level of significance after Bonferroni correction was *p* < 0.025.

##### Average weekly global scores

The average weekly global score (AWG) (maximum score 105) was calculated for each dog using the following equation:

$$ AWG=\frac{Sum\  of\  all\  AWR\  that\  week}{Number\  of\  eliciting\  contexts\  recorded\  that\  week} $$

To compare the average weekly global scores in dogs between baseline and specific time points (week 1, week 11, decision point) whilst on treatment through the study, paired sample t-tests were used. The accepted level of significance after Bonferroni correction was *p* < 0.025.

#### Effects on temperament

##### Positive and negative activation

To compare the effects of treatment on owner reported positive and negative activation (from the PANAS) between baseline and the decision point of treatment, paired sample t-tests were used. A value of *p* < 0.05 was considered significant.

### Owner satisfaction with treatment

A Spearman’s correlation coefficient was computed to assess whether owner satisfaction with treatment success correlated with: i) changes in reactions (percentile reduction in average global weekly fear-anxiety reactions from baseline to decision point); ii) changes in temperament (percentile reduction in negative activation from baseline to decision point). A value of *p* < 0.05 was considered significant.

At the end of the study, owners were also asked about their interest in continuing treatment with imepitoin at their own expense and to rate the ease of administration of the imepitoin

## Results

### Demographics

The initial group was composed of 20 dogs (see Table 2): three dogs were withdrawn from the study: case 1 due to change in analgesia during the follow-up period whilst on imepitoin case 4 due to a reported adverse event whilst on imepitoin; case 18 due to abnormalities in initial laboratory evaluation results before imepitoin was commenced. This left 17 dogs in the final analysis (Table 3) comprising 11 females (64.7%) and 6 males (35.3%) (all dogs were neutered), of a range of breeds and ages ranging from 1 year 1 month to 10 years 7 months (average age of 4 years 6 months). Bodyweight ranged from 5.0 kg to 36.7 kg (average bodyweight 18.9 kg). All dogs had been owned for a minimum of 3 months by their owners prior to enrolment.

**Table 2:** Initial group and overview of clinical details and withdrawals

Case No.	Breed	Sex^a^	Age at clinic visit	Weight (kg)	Eliciting contexts recorded	Other treatments	Notes on other treatments	Adverse events	Reason for withdrawal
1	Cross-breed	MN	3 y 8mo	21.00	Noises; Carrying objects; Entering back door	Meloxicam SID	Changed to firocoxib during study	Y	Change of analgesia
2	Cross-breed	FN	6y	11.60	Noises	Carprofen SID	Continued during study	N	N/A
3	Cross-breed	MN	3y 7mo	21.30	Noises	None	-	N	N/A
4	Labrador Retriever	FN	7y	23.75	Noises; Visitors; Dogs	None	-	Y	Adverse events
5	Staffordshire Bull Terrier	FN	10y 7mo	18.45	Noises	Meloxicam SID	Trialled only, not used during study	N	N/A
6	Whippet	FN	3y 5mo	12.74	Noises; Walking	None	-	N	N/A
7	Staffordshire Bull Terrier	FN	2y 5mo	18.98	Noises; Walking	None	-	Y	N/A
8	Brittany Spaniel	FN	8y 4mo	12.78	Noises; Walking	None	-	N	N/A
9	English Bull Terrier	FN	2y 8mo	20.30	Visitors; Strangers; Noises; Moving from sofa	Firocoxib SID	Trialled only, not used during study	N	N/A
10	Labrador Retriever	MN	3y 9mo	25.75	Noises	None	-	N	N/A
11	German Wire-haired Pointer	MN	4y 1mo	31.60	Novel items; Strangers; Visitors; Postal delivery; Noises	Meloxicam SID	Trialled only, not used during study	Y	N/A
12	Tervuren Belgian Shepherd	MN	5y 1mo	23.40	Noises; Walking	Carprofen SID	Trialled only, not used during study	N	N/A
13	Cross-breed	FN	4y 10mo	20.20	Noises; Visitors	None	-	N	N/A
14	Cross-breed	FN	1y 1mo	20.00	Visitors; Strangers; Crowded areas; Dogs; New environments	None	-	N	N/A
15	Jack Russell Terrier	FN	5y	7.36	Noises; Walking	None	-	N	N/A
16	Cross-breed	FN	2y 3mo	18.48	Noises	None	-	N	N/A
17	Cross-breed	FN	2y 9mo	5.00	Home alone	None	-	Y	N/A
18	Border Collie	FN	6y 7mo	12.86	Noises	Firocoxib SID	-	N/A	Blood results
19	Cross-breed	MN	1y 7mo	15.40	Dogs; Strangers; Visitors	None	-	N	N/A
20	German Shepherd	MN	5y 1mo	36.70	Noises	Meloxicam SID	Continued during study	Y	N/A

^a^ *M* male, *F* female, *N* neutered, *y* years, *mo* months, *Y* yes, *N* no, *N/A* not applicable

**Table 3:** Population used in final analysis, dose of imepitoin, AWG and PANAS at specific time points and owner satisfaction

Case No.	Baseline		Week 1		Week 11		Decision		Max. dose of imepitoin (mg/kg BID)	Score Reduction Negative Activation (Baseline to Decision)	% Score Reduction Negative Activation (Baseline to Decision)	Owner Satisfaction	Continuation of treatment at owner’s expense – Y/N (C/T) = basis of use
	AWG	Dose (mg/kg)	AWG	Dose (mg/kg)	AWG	Dose (mg/kg)	AWG	Dose (mg/kg)					
2	46.70	0	26.50	8.6	9.00	30.2	4.00	30.2	30.2	−0.05	−6.25	S	Y (C)
3	11.57	0	2.50	9.4	0.50	19.8	2.50	19.8	19.8	−0.17	−22.67	S	Y (T)
5	32.33	0	24.50	10.8	11.00	21.6	0.00	21.6	21.6	−0.38	−47.50	VS	Y (C)
6	3.36	0	0.88	11.8	0.00	3.9	0.00	11.8	11.8	−0.04	−4.76	VS	Y (T)
7	20.94	0	4.36	10.5	5.00	10.5	5.00	10.5	10.5	−0.36	−46.15	VS	Y (C)
8	19.75	0	3.33	11.7	0.59	11.7	0.00	23.5	23.5	−0.04	−6.25	S	Y (T)
9	12.00	0	7.33	9.9	3.34	9.9	6.00	19.8	19.8	−0.18	−30.14	S	Y (C or T)
10	23.67	0	27.50	9.7	6.67	19.3	6.50	19.3	19.3	−0.16	−22.22	VS	Y (T)
11	28.07	0	13.38	12.7	9.00	19.0	9.67	19.0	19.0	−0.15	−21.13	S	Y (T)
12	34.75	0	34.00	10.7	9.64	19.2	17.58	29.9	29.9	−0.07	−8.54	PP	Y (C)
13	29.75	0	3.50	9.9	16.67	19.8	2.50	29.7	29.7	−0.31	−34.07	S	Y (C or T)
14	28.58	0	12.78	10.0	11.75	20.0	5.50	20.0	20.0	−0.15	−17.24	PP	N
15	34.91	0	21.00	13.6	8.00	30.5	1.00	30.5	30.5	−0.07	−8.54	VD	N
16	7.61	0	4.59	10.8	7.00	29.8	7.00	29.8	29.8	0.05	9.80	PP	N
17	28.00	0	21.00	10.0	1.00	5.0	1.00	5.0	10.0	−0.03	−5.88	VS	Y (C)
19	28.89	0	19.84	9.7	16.33	9.7	15.50	19.4	19.4	−0.16	−18.18	VS	Y (C or T)
20	6.33	0	3.33	10.9	8.00	21.8	9.50	21.8	21.8	−0.22	−30.14	PP	N

*AWG* Average weekly global score, *Baseline* Baseline week, *Decision* Decision point (11–19 weeks); *Y* yes, *N* no, VS very satisfied, *S* satisfied, *PP* partly satisfied/partly dissatisfied, *D* dissatisfied, *VD* very dissatisfied, *C* continuous use, *T* tactical use

### Clinical examination and laboratory evaluation findings

Clinical examination revealed abnormalities in 6 cases, with some cases having more than 1 abnormality: gait abnormalities/musculoskeletal problems (5 dogs), interdigital cyst (1 dog), entropion (1 dog), dental disease (1 dog). Further investigations/treatments were performed at the referring veterinary practices. Where this included the use of trial analgesia, or surgical correction (canine extraction, entropion correction) a new baseline week of diary entries was established after recovery/once deemed stable on analgesia for approximately 4 weeks. Laboratory evaluation revealed abnormalities in one dog (case 18) indicative of atypical hypoadrenocorticism and this dog did not proceed to the treatment group.

### Adverse events

Among the 19 dogs treated with imepitoin, 7 adverse events were reported in 6 dogs, these were not necessarily associated with medication but are recorded here in accordance with best practice. Two dogs showed signs of ataxia, and 1 dog chewed some plastic and vomited. These three incidents resolved without specific treatment whilst continuing the imepitoin. Recurrence of a pre-existing lameness occurred in Case 1 resulting in withdrawal from the study by the investigators due to a change in analgesia regime by the referring veterinarian. Diarrhoea was seen in 1 dog (Case 17) which resolved on cessation of imepitoin at 10 mg/kg; when the drug was reintroduced at the lower dose of 5 mg/kg there was no recurrence in diarrhoea and the dog remained on this dose. Case 4 had 2 episodes of ataxia and muscle tremors, (including around the head) the first during week 7 and the second during week 8 of treatment with 10 mg/kg imepitoin. Both of these episodes occurred around the time where noises were audible which had historically resulted in a fearful response. The blood profile was repeated and no abnormalities were detected. Despite general improvements in behaviour in response to noise out with these events, the owner opted to stop the imepitoin and the dog was withdrawn from the study.

### Fear-anxiety triggers

Of the 17 cases used in the final analysis 6 cases (35.3%) had a single eliciting context monitored, with the remaining 11 cases (64.7%) having 2 or more eliciting contexts each. This gave a total of 35 different eliciting contexts for fear-anxiety: 13 social (visitors, strangers, dogs, crowded areas, home alone) and 22 non-social (14 of which were noises, the remainder being: walking, moving from sofa, novel items, postal delivery, new environments).

### Behaviour modification programmes

Behaviour modification recommendations generally included managing the dog’s exposure to triggers which included environmental modifications and reinforcing appropriate behaviour. Other recommendations included introduction of a safe haven at home (12 cases); operant counterconditioning protocols with hand-touch to check-in with owner when the dog has heard a noise on walks (10 cases); desensitisation and counterconditioning protocols with sound CDs (9 cases – only 1 of the 9 used the sound CD during the follow up period); basket muzzle training (4 cases); teaching a ‘go to mat’ behaviour (3 cases); introduction of activity feeders (3 cases); introduction of front-attaching harness (2 cases); desensitisation and counterconditioning to vet practice (1 case); toilet training advice (1 case); cues for ‘say hello, say goodbye’ when meeting and disengaging from other dogs (1 case). The single case of separation related problems was advised on desensitisation to leaving rituals, introduction of safety signal when leaving dog alone, changing leaving/returning ritual and teaching increased independence from owner.

### Use of imepitoin

During the study, of the 17 dogs in the final analysis, 4 received a maximum trial dose of around 30 mg/kg twice daily, 10 dogs received a maximum trial dose of around 20 mg/kg twice daily and 2 dogs remained on the initial dose of around 10 mg/kg twice daily, with 1 dog dropping down to and being maintained on 5 mg/kg twice daily. Mean doses of imepitoin being used at key time points were: week 1 (mean = 10.6 mg/kg, median = 10.5 mg/kg); week 11 (mean = 17.7 mg/kg, median = 19.3 mg/kg); decision point (mean = 21.3 mg/kg, median = 20.0 mg/kg).

### Effects on reactions

#### Average weekly global scores

The primary measure of interest was the average weekly global scores (AWG). An Anderson-Darling normality test showed that the data distribution was not significantly different from normal across the 17 subjects at baseline (*p* = 0.306), week 11 (*p* = 0.463), decision point (*p* = 0.088) and mean (*p* = 0.064), but were significantly different to normal at week 1 (*p* = 0.037). In this situation, parametric tests can be used to examine effects within the population studied, but generalisation to the wider population should be more cautious [44] for the data relating to non-normally distributed data. Given the acknowledged preliminary nature of this study (case series), it is therefore acceptable to use parametric tests with this caution acknowledged. Summary effects are displayed in Table 4.

**Table 4:** Average weekly global fear-anxiety reaction scores for all 17 dogs in final analysis

Week	Average weekly global fear-anxiety reaction score			t-value (16)	*p*-value	d_Cohen_
	Mean +/− SD	Decrease from baseline (95% CI)	Median (range)			
Baseline	23.37 +/− 11.87	-	28.00 (3.36–46.70)	-	-	-
1	13.55 +/− 10.75	9.82 (5.76,13.88)	12.78 (0.88–34.00)	5.13	<0.001	−0.867
11	7.26 +/− 5.13	16.10 (10.88,21.32)	8.00 (0–16.67)	6.54	<0.001	−1.762
Decision	5.49 +/− 5.21	17.88 (11.48,24.28)	5.00 (0–17.58)	5.92	<0.001	−1.951

There was a statistically significant difference in the AWG between baseline and all specific time points whilst on treatment. These effect sizes are large and the results indicate that imepitoin alongside a behaviour modification programme had a useful effect on reducing AWG in dogs, with an initial meaningful effect being seen within the first week of treatment.

#### Average weekly reaction scores

The secondary measure of interest was the average weekly reaction scores (AWR) between the eliciting context groups previously described. An Anderson-Darling normality test showed that the data were normally distributed across the average weekly reactions for different eliciting contexts and subjects at baseline (*p* = 0.565), week 1 (*p* = 0.051) and week 11 (*p* = 0.139), but not at the decision point (p = <0.005).

##### Noise sensitive group

For the noise sensitive group (*n* = 14), there was a significant difference in the AWR between: baseline and week 1; baseline and week 11; baseline and decision point. There was a larger effect size at week 11 and decision point compared to week 1. The data are displayed in Table 5.

**Table 5:** Average weekly reaction score – noise sensitive group (*n* = 14)

Week	Noise sensitivities - Average weekly reaction score			t-value (13)	*p*-value	d_Cohen_
	Mean +/− SD	Decrease from baseline (95% CI)	Median (range)			
Baseline	21.11 +/− 13.50	-	21.50 (4.92–46.70)	-	-	-
1	14.17 +/− 11.65	6.94 (3.07,10.81)	10.50 (0.25–34.00)	3.87	0.002	−0.5504
11	8.30 +/− 6.71	12.81 (6.55,19.08)	7.00 (0.50–26.50)	4.42	0.001	−1.2017
Decision	8.26 +/−11.11	12.85 (5.10,20.59)	5.75 (0.00–43.00)	3.58	0.003	−1.039

##### Non-social fear-anxiety group

For the non-social fear-anxiety group (excluding noise sensitivities) (*n* = 8), there was a significant difference in the average weekly reaction scores between: baseline and week 1; baseline and week 11; baseline and decision point. There was a larger effect size at week 11 and decision point compared to week 1. The data are displayed in Table 6.

**Table 6:** Average weekly reaction score – non-social fear/anxiety group (excluding noise sensitivities) (*n* = 8)

Week	Non-Social - Average weekly reaction score			t-value (7)	*p*-value	d_Cohen_
	Mean +/− SD	Decrease from baseline (95% CI)	Median (range)			
Baseline	18.57 +/− 10.20	-	19.02 (1.79–30.00)	-	-	-
1	9.76 +/− 6.59	8.81 (2.03,15.60)	10.17 (0.00–20.00)	3.07	0.018	−0.782
11	6.78 +/− 5.56	11.79 (6.07,17.51)	7.88 (0.00–15.00)	4.88	0.002	−2.786
Decision	6.51+/− 5.57	12.06 (4.75,19.38)	6.88 (0.00–15.00)	3.90	0.006	−2.824

##### Social fear-anxiety group

For the social fear-anxiety group (*n* = 13), there was a significant difference in the average weekly reaction scores between: baseline and week 1; baseline and week 11; baseline and decision point. There was a larger effect size at week 11 and decision point compared to week 1. The data are displayed in Table 7.

**Table 7:** Average weekly reaction score – social fear/anxiety group (including single case of separation related problems) (*n* = 13)

Week	Social - Average weekly reaction score			t-value (12)	*p*-value	d_Cohen_
	Mean +/− SD	Decrease from baseline (95% CI)	Median (range)			
Baseline	25.68 +/− 7.82	-	27.20 (13.00–38.00)	-	-	-
1	15.48 +/− 8.97	10.20 (7.15,13.25)	19.00 (1.33–26.25)	7.28	<0.001	−1.112
11	8.48 +/− 6.11	17.20 (12.81,21.59)	7.00 (0.00–17.00)	8.54	<0.001	−2.451
Decision	8.22 +/− 6.38	17.46 (12.73,22.20)	7.00 (0.00–19.33	8.03	<0.001	−2.447

These results indicate large effects in reducing average weekly reaction scores across dogs with noise sensitivities, social and non-social fears and anxieties, with significant effects being seen within the first week across all groups, and larger effects being seen at week 11 and decision points compared to week 1.

### Effects on temperament

#### Negative activation

The paired sample t-test used to compare negative activation scores showed a significant difference in the mean scores between baseline and the decision point with a large effect size.

#### Positive activation

There was no significant difference in the mean positive activation scores between baseline and the decision point. Effect size was therefore not calculated. The data are displayed in Table 8.

**Table 8:** Positive and negative activation scale scores

Week	Baseline Mean +/− SD	Baseline Median (range)	Decision Point Mean +/− SD	Decision Point Median (range)	Mean Decrease from baseline (95% CI)	t-value (16)	*p*-value	d_Cohen_
Negative activation	0.7541 +/− 0.1148	0.78 (0.51–0.91)	0.6053 +/− 0.1216	0.58 (0.42–0.80)	0.1488 (0.0865,0.2111)	5.07	<0.001	−1.258
Positive activation	0.6653 +/− 0.1152	0.70 (0.52–0.84)	0.6665 +/− 0.1236	0.66 (0.52–0.87)	0.0012 (0.0303,0.0280)	−0.09	0.933	-

These results suggest that imepitoin alongside a behaviour modification programme has a large statistically significant effect on reducing negative activation (i.e. fearfulness/anxiousness in dogs) as measured through the PANAS.

### Owner satisfaction with treatment

Owners were also asked to rate their overall satisfaction with treatment success: 6 (35.3%) were very satisfied, 6 (35.3%) satisfied, 4 (23.5%) partly satisfied/partly dissatisfied, 0 dissatisfied and 1 (5.9%) very dissatisfied. The owner who was very dissatisfied (case 15) reported an improvement in anxiety on walks during treatment, but insufficient change in reactions to noises which was the main presenting problem.

There was no correlation between owner satisfaction with either percentile reduction in average global weekly fear-anxiety reactions (*r*_*s*_ = −0.288; *p* = 0.262) or percentile reduction in negative activation from baseline to decision point. (*r*_*s*_ = −0.201; *p* = 0.439).

### Continuation of treatment at owners’ expense

Of the 17 owners, 13 (76.5%) suggested they would use imepitoin at their own expense: 5 opted to use it on a continual basis from the end of the follow up period; 5 suggested they would consider using it on a tactical basis (e.g. leading up to and for the duration of periods during the year with increased risk of exposure to stressors); 3 suggested they would use it either continually or tactically depending on the outcome of discontinuing the imepitoin on their dog’s behaviour – i.e. if improvements seen during the treatment period were not sustained, they would consider restarting the use of imepitoin. The remaining 4 owners (23.5%) suggested they would not consider using imepitoin again. In 3 of the 5 cases who opted to stop imepitoin on a continual basis and consider use on a tactical basis, the consulting clinician (KM) would have preferred using the imepitoin on a continuous basis given the reported improvements during the follow up period. In all other decisions the clinician was in complete agreement with the client’s decision.

### Ease of administration

At the end of the study, owners were asked to rate the ease of administration of imepitoin with the results being: 6 (35.3%) very easy, 9 (52.9%) easy, 2 (11.8%) moderately easy/moderately difficult. No owners rated administration as difficult or very difficult.

### Additional information

#### Owner reported improvements with change in dose of imepitoin

During the study, there were 7 owner reports of improvements in behaviour quickly following dose increases in imepitoin suggesting specific efficacy of imepitoin. Five cases where dose was increased from approximately 10 mg/kg to 20 mg/kg (Cases 5, 11, 12, 13, 16), and 2 cases where dose was increased from around 20 mg/kg to around 30 mg/kg (Cases 2, 12).

#### Owner reported reduction in recovery time during treatment with imepitoin

Three owners (Case 5, 12, 13) reported reduction in recovery time (i.e. quicker recovery) whilst on imepitoin following exposure to fireworks compared to previous years.

#### Owner reported deterioration on cessation of imepitoin

Two owners continued to keep diary entries through weaning, and behavioural deterioration on cessation of therapy with continuation of the behaviour modification programme suggests specific efficacy of imepitoin. In case 15 the owner reported an increase reluctance to go for walks which had improved during the period on imepitoin. In Case 3 the owner reported an increase in noise sensitivity and reactivity to other dogs at classes which had improved during the period on imepitoin.

### Dog’s responses from diaries

A total of 1210 individual diary forms were completed by owners during the study. Dog’s responses for each of the options from the diary were assessed with mean scores between baseline (‘off imepitoin) and the entire treatment period (‘on imepitoin’) with the percentage change shown in Table 9. There was a reduction in mean scores of all clinical signs across all eliciting contexts during treatment compared with baseline with the exception of one single episode of diarrhoea (under title of ‘vomiting/urinating/defecating’) when home alone reported for Case 17 which was reported as an adverse event.

**Table 9:** Clinical signs as reported by owners across all eliciting contexts between baseline and whole period on imepitoin

Dog’s response from eliciting context diary	Mean scores at baseline	Mean scores on imepitoin	% change in scores
Running around	0.802	0.412	−48.6
Drooling saliva	0.481	0.165	−65.7
Hiding	1.091	0.577	−47.1
Destructiveness	0.092	0.017	−81.5
Cowering	2.588	1.096	−57.7
Restlessness/pacing	1.344	0.570	−57.6
Aggressive behaviour	0.310	0.154	−50.3
“Freezing” to the spot	1.114	0.459	−58.8
Barking/whining/howling	0.786	0.608	−22.6
Panting	1.605	0.772	−51.9
Vomiting/defecating/urinating	0.000	0.005	+0.50
Owner-seeking behaviour	1.277	0.750	−41.3
Vigilance/scanning of environment	2.128	1.406	−33.9
Bolts	0.939	0.460	−51.0
Exaggerated response when startled	2.273	1.063	−53.2
Shaking or trembling	1.567	0.482	−69.2
Self-harm	0.017	0.007	−58.8
Yawning	0.522	0.249	−52.3
Licking lips	1.693	0.683	−59.7
Moving away	1.661	0.477	−71.3

## Discussion

These results provide initial evidence of the potential value of using imepitoin alongside a behaviour modification programme in reducing the average weekly global scores reported by owners of dogs with fear-anxiety related problems. When grouped into noise sensitivity, social and non-social fear-anxiety based problems, a reduction in average weekly reaction scores were seen across all groups. These effects appeared to be quick acting (within a week of the onset of administration of treatment) but improve over time. In addition imepitoin alongside a behaviour modification programme significantly reduced owner report of fearfulness as a temperament trait in dogs. Therefore it may be of value in cases where dogs have been identified as having a generally fearful or anxious temperament and potentially to help prevent a recurrence of problems in such individuals, although this remains to be tested empirically. At the end of the study 76.5% of owners would consider using imepitoin at their own expense, 88.2% of owners found imepitoin very easy or easy to administer, and 70.6% of owners were satisfied or very satisfied with treatment success. This demonstrates that there were also clinically meaningful improvements being seen by dog owners during the treatment period, although owner satisfaction did not relate to the scale of improvement measured here. This might be because owner satisfaction may depend on a few signs that are of particular concern to them (e.g. destructiveness or vocalisation) [10] whereas the metric used to assess improvement considers all signs even if not problematic to the owner.

Although case series provide a relatively low level of evidence among clinical studies [45] they can serve an important purpose in advancing medical knowledge [46]; for example, this report provides the first exploration of the potential application of imepitoin as an anxiolytic for clinical behaviour problems. The majority of dogs had multiple problems extending from fear-anxiety, and so two types of metric were used. One type of metric (average global weekly scores; negative activation scores) gave a measure of the overall response for the totality of problems. The other (average weekly reaction scores) focused on the response to treatment to particular types of problem, grouped by context into social, non-social or noise related fear-anxiety problems. This resulted in dogs being included in multiple evaluations for average weekly reaction scores according to which context(s) the problem(s) were apparent, however only data relating to the relevant eliciting contexts for that context were included in that assessment, and not the global problem data.

In the current study, baseline diary entries before commencing treatment meant each dog could acted as its own control, and although there was no control for dogs receiving imepitoin without behaviour modification advice, this is more typical of the recommended way of using medication in clinical behaviour cases. Medication is typically used as an adjunct to behaviour modification advice therefore this study replicates how imepitoin would likely be used in veterinary behavioural practice. However, given the rapid onset of effect within the first week, the finding that during the study there were 7 owner reports of improvements in behaviour quickly following dose increases, and the 2 owner reports of deterioration in their dogs behaviour on cessation of imepitoin, it seems likely that there is a specific effect of the drug in these cases and the results are not simply due to the behaviour modification programme.

These improvements seen within the first week of treatment, also raise the question of the potential to use imepitoin tactically. In the current study, this was important for those owners who saw benefits whilst their dog was on imepitoin but did not wish to use it continuously, but would consider using it at times of the year where there would be an increased risk of exposure to triggers for fear-anxiety (e.g. fireworks season, shooting season). For these cases it was advised to use imepitoin, in future, at the dose found to be effective during the current study, commencing 1 week before the anticipated onset of problematic triggers, and for the duration of this period (this could range from days to weeks depending on the trigger and level of exposure in the environment anticipated by the owner for the dog). Currently unlicensed benzodiazepines are often used tactically, given 30–60 min before the fear-anxiety trigger is expected, but responses to medication by individuals are often hard to predict [24, 25]. Further studies are required to investigate the potential benefits of using imepitoin in this way or in the context of individual dosing in relation to a specific event, in particular, the minimum period of time imepitoin can be given for an effect to be seen. Typically other anxiolytics such as SSRIs and TCAs may have an onset of action of around 3–5 weeks [15], so in cases where a more rapid onset of action is required, imepitoin may be a valuable option.

There were significant differences in reports of reactions to noises between week 1 of treatment and both week 11 and decision point. It should be noted that the average dosage of imepitoin being administered across all dogs differed at these time points, as it was determined and increased on an individual basis. Mean dosage at week 1 was 10.6 mg/kg, at week 11 was 17.7 mg/kg, and at the decision point was 21.3 mg/kg. Reduction in reaction scores could therefore reflect a difference in dose being given, be the result of a longer duration of treatment or reflect the synergistic effect of imepitoin and behaviour modification therapy. However, in light of these findings 20 mg/kg imepitoin twice daily appears to be a reasonable recommended dose for anxiolytic effects with dose titration built around this dose according to effect.

The study used a standardised diary format to reduce subjectivity in owner responding, and self-reporting to gather data for analysis. There can be multiple limitations with this method - during the study there were 3 owners who suggested that they were failing to record all eliciting contexts resulting in ‘no response’ from their dog. This suggests that improvements may actually have been under-reported. The diary was used to record a summary of what the owner saw during the whole event their dog was exposed to the eliciting context, and there was no standardised duration of time each owner was required to observe their dog for. A caregiver placebo effect has been shown in dogs receiving treatment for osteoarthritis [47] and should also be considered when assessing the data from the study, which can potentially result in over-reporting of improvements by owners. The diary used did not include a section on recovery time after exposure to an eliciting context however 3 owners spontaneously reported reduced recovery times after exposure to noises compared to the time before treatment with imepitoin. Recovery time might therefore be a useful metric to include in future studies.

There were 6 dogs with 7 reported adverse events during the trial, with a high likelihood (as judged by the authors) of the imepitoin being implicated in 2 of these (mild ataxia, Case 11; diarrhoea, Case 17) but the likelihood of the drug’s involvement in the other 3 is largely unknown although in one we consider it unlikely (Case 1).

Paradoxical excitement can occur in species (including dogs) receiving benzodiazepines [24, 31], and although a potential adverse effect of using imepitoin is transient hyperactivity [35], there were no dogs in the study where this was reported.

Tolerance to the effects of benzodiazepines can develop with ongoing use [15, 31] such as those demonstrated in anxiolytic models in mice [48]. The development of tolerance to the anticonvulsant effects of diazepam in dogs has also been documented [49]; however tolerance to the anticonvulsant effects of imepitoin has not been demonstrated [33, 37]. Additionally, when looking at anxiolysis, the results of this study showed significant effects within the first week of treatment with continued improvements seen at week 11 and decision points which is not suggestive of tolerance developing within this time frame.

Concerns have been raised over the potential for benzodiazepines disinhibiting and therefore increasing aggressive behaviour in dogs and other species [15, 31] and one study reported increased aggressive behaviour and new aggression in a small proportion of dogs treated with diazepam [24]. There were no dogs in the study where aggressive behaviour was reported in the diary entries where it had not been seen before, and overall, there was a 50.3% reduction in owner reported signs of aggressive behaviour across all dogs whilst on imepitoin compared to the baseline period.

In the human literature, there is conflicting evidence supporting or refuting the amnesic, memory and cognitive impairing effects of long term benzodiazepine use (for a meta-analysis and review see [50]). During the study there were no reports of adverse events suggestive of memory impairments such as loss of previously learned behaviours. In addition, all owners were asked to use operant and/or classical conditioning as part of their individualised behaviour modification programmes and subjectively there appeared to be no difficulty in implementation of such training protocols.

It is important to consider both the physical health and concurrent medical problems of any patient presenting with a behavioural complaint [51]. Although this study excluded dogs with a history of seizures, given that behavioural co-morbidities are common in canine epilepsy [38], further studies could explore the value of using imepitoin in these patients. Musculoskeletal pain may cause or exacerbate behaviour problems such as aggressive behaviour [52, 53]. Fear and anxiety may also amplify pain [54]. For these reasons, it was important to establish that any medical abnormalities detected on clinical examination were investigated and treated prior to commencing imepitoin and a behaviour modification plan, which occurred in 6 of the 20 dogs enrolled in the study. Indeed 11 subjects were not enrolled in the study due to uncontrolled medical reasons, with 8 of these related to suspected musculoskeletal pain. In addition one dog (Case 1) was withdrawn from the analysis due to recurring lameness which resulted in increased general anxiety and required a change in analgesia. Pain remains an important differential and moderator of response in anxiety cases, and so all cases need careful medical evaluation.

## Conclusions

This report provides the first exploration of the potential application of imepitoin as an anxiolytic for clinical behaviour problems. This study suggests that imepitoin may be an effective medication to aid the treatment of a range of fear-anxiety based problems when used alongside a behaviour modification programme. The current veterinary formulation of imepitoin licensed for long term use in dogs with epilepsy appears to be safe and well tolerated. A dose of 20 mg/kg imepitoin twice daily appears to be an appropriate starting dose for anxiolytic effects in most patients, with a fast onset of action with improvements being seen within the first week of commencing treatment, giving it an advantage over some other classes of anti-anxiety medication such as TCAs and SSRIs. Further research with a randomized, double-blind, placebo-controlled trial would be useful to confirm the apparent efficacy reported here.

## Additional file

Additional file 1: Eliciting context diary. Owner diary used during study to record their dog’s behaviour during exposure to an eliciting context. (DOCX 64 kb)
