# Supplementary material for: The use of imepitoin (Pexion™) on fear and anxiety related problems in dogs – a case series
Source: BMC Vet Res. 2017 Jun 13;13:173. doi: 10.1186/s12917-017-1098-0 (PMC5470190; doi:10.1186/s12917-017-1098-0)
Supplement: Additional file 1: — Eliciting context diary. Owner diary used during study to record their dog’s behaviour during exposure to an eliciting context. (DOCX 64 kb) [file 12917_2017_1098_MOESM1_ESM.docx]

**ADDITIONAL FILES**

*Additional file 1: Eliciting context diary* **
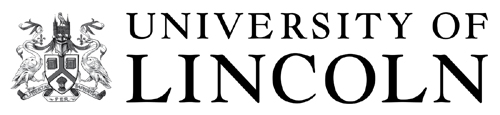
**

**Imepitoin Study Diary – Baseline Information**

**Patient: Case number:**

**Eliciting Context Summary**

1. **Eliciting Context EC1:**
2. **Eliciting Context EC2:**
3. **Eliciting Context EC3:**
4. **Eliciting Context EC4:**

**Instructions for Completing the Table**

Before treatment is commenced, we need some baseline information about your dog’s normal reaction to the eliciting contexts we have identified. Each time that one of these eliciting contexts occurs please record the following details (whether there is a reaction or not):

- The date that the context has occurred-this is to be recorded in the format DD/MM (e.g. 12^th^ November = 12/11);
- The eliciting context number (e.g. EC1) or a description of the event if you are not sure;
- Under ‘Event occurrence’ tick either ‘Yes’ or ‘No’ to indicate your dog’s response in relation to this measure. Please do NOT leave both of them blank, as we need you to confirm one way or the other each sign.
- If a response was seen which is not included in the table, please add it in at ‘21. Others’
- Under ‘Severity’ please grade this 1-5;

The ‘Score’ column is for office use only, so please leave this blank

**Date:** _ _/_ _ (DD/MM)

***Eliciting context* number** - EC**__**

| **Dog’s**  **Response** | **Event occurrence** | | **Severity** | | | | | **Score**  *(for office use only)* |
| --- | --- | --- | --- | --- | --- | --- | --- | --- |
|  | **Yes** | **No** | **1** | **2** | **3** | **4** | **5** |  |
| 1.Running around |  |  | Small amount – occasional burst of activity |  |  |  | Extensive amount – continuously running around |  |
| 2. Drooling saliva |  |  | Small amount – damp around mouth |  |  |  | Extensive amount – pools of saliva |  |
| 3. Hiding (e.g. under furniture, behind owner etc.) – please indicate where:  _________________________ |  |  | Small amount - retreats |  |  |  | Extensive amount – will not be moved from hiding area |  |
| 4. Destructiveness (e.g. furniture, doors, carpets, etc) – please indicate which items tend to be damaged:  _________________________ |  |  | Small amount – small items, e.g. pens |  |  |  | Extensive amount – e.g. holes in wall |  |
| 5. Cowering (e.g. tucks tail, flattens ears, etc) |  |  | Small amount - uneasy |  |  |  | Extensive amount – petrified |  |
| 6. Restlessness/pacing |  |  | Small amount |  |  |  | Extensive amount – fixed route continuously traced |  |
| 7. Aggressive behaviour (e.g. growling, snarling, snapping, biting) – please indicate which of these:  _________________________ |  |  | Small amount – occasional growl |  |  |  | Extensive amount – severe biting attempts made |  |
| 8. “Freezing” to the spot |  |  | Occurs sporadically within the event |  |  |  | Most of the time during the event |  |
| 9. Barking/whining/howling – please indicate which of these:  _________________________ |  |  | Small amount |  |  |  | Extensive amount |  |
| 10. Panting |  |  | Occurs sporadically within the event |  |  |  | Most of the time during the event |  |
| 11.Vomiting/defecating/  urinating and/or diarrhoea – please indicate which of these:  _________________________ |  |  | Small amount |  |  |  | Extensive amount |  |
| 12. Owner-seeking behaviour |  |  | Seeks out owner occasionally during the event |  |  |  | Will not leave owner in any circumstance |  |
| 13. Vigilance/scanning of environment |  |  | Occurs sporadically within the event |  |  |  | Most of the time |  |
| 14. Bolts |  |  | Occurs then seems to settle |  |  |  | Occurs frequently and cannot settle in between |  |
| 15. Exaggerated response when startled |  |  | Occurs then seems to settle |  |  |  | Remains very jumpy throughout |  |
| 16. Shaking or trembling |  |  | Slight tremble / twitching |  |  |  | Severe shaking throughout |  |
| 17. Self-harm |  |  | Small amount – e.g. licking feet |  |  |  | Extensive amount – e.g. serious wound |  |
| 18. Yawning |  |  | Single event |  |  |  | Frequent during the event, before and afterwards |  |
| 19. Licking lips |  |  | Single event |  |  |  | Frequent during the event, before and afterwards |  |
| 20. Moving away |  |  | Occurs then seems to settle |  |  |  | Occurs frequently and cannot settle in between |  |
| 21. Others – please detail:  _________________________ |  |  | Small amount |  |  |  | Extensive amount |  |

Total score = _____
